# A Multiple Health Behavior Change, Self-Monitoring Mobile App for Adolescents: Development and Usability Study of the Health4Life App

**DOI:** 10.2196/25513

**Published:** 2021-04-12

**Authors:** Louise Thornton, Lauren Anne Gardner, Bridie Osman, Olivia Green, Katrina Elizabeth Champion, Zachary Bryant, Maree Teesson, Frances Kay-Lambkin, Cath Chapman, The Health4Life Team

**Affiliations:** 1 The Matilda Centre for Research in Mental Health and Substance Use The University of Sydney Sydney Australia; 2 Priority Research Centre for Brain and Mental Health The University of Newcastle Newcastle Australia; 3 School of Public Health and Community Medicine University of New South Wales Sydney Australia; 4 Department of Exercise Physiology School of Medical Sciences, Faculty of Medicine University of New South Wales, Sydney Sydney Australia; 5 Department of Preventive Medicine Northwestern University Feinberg School of Medicine Chicago, IL United States; 6 National Drug Research Institute Curtin University Perth Australia; 7 School of Psychology Centre for Youth Substance Abuse Research The University of Queensland Brisbane Australia

**Keywords:** mHealth, mobile phone, chronic disease, adolescents, health promotion

## Abstract

**Background:**

The link between chronic diseases and the Big 6 lifestyle risk behaviors (ie, poor diet, physical inactivity, smoking, alcohol use, sedentary recreational screen time, and poor sleep) is well established. It is critical to target these lifestyle risk behaviors, as they often co-occur and emerge in adolescence. Smartphones have become an integral part of everyday life, and many adolescents already use mobile apps to monitor their lifestyle behaviors and improve their health. Smartphones may be a valuable platform for engaging adolescents with interventions to prevent key chronic disease risk behaviors.

**Objective:**

The aim of this paper is to describe the development, usability, and acceptability of the Health4Life app, a self-monitoring smartphone app for adolescents that concurrently targets the Big 6 lifestyle behaviors.

**Methods:**

The development of the Health4Life app was an iterative process conducted in collaboration with adolescents and experts. The development process consisted of three stages: scoping the literature; end user consultations, which included a web-based survey (N=815; mean age 13.89, SD 0.89 years) and a focus group (N=12) among adolescents; and app development and beta testing. Following this development work, 232 adolescents were asked to rate the usability and acceptability of the app.

**Results:**

The process resulted in a self-monitoring smartphone app that allows adolescent users to track and set goals for the Big 6 health behaviors, using in-app rewards and notifications to enhance engagement. The overall adolescent feedback was positive in terms of user-friendly design, content, relevance, and helpfulness. Commonly identified areas for improvement were to increase interactive features and display recorded health behaviors differently to improve interpretability.

**Conclusions:**

The Health4Life app is a co-designed, self-monitoring smartphone app for adolescents that concurrently targets the Big 6 lifestyle behaviors. Adolescents rated the app as highly acceptable and usable. The app has the potential to efficiently and effectively modify important risk factors for chronic disease among young people and is currently being evaluated in a world-first trial of 6640 secondary school students in 71 schools across Australia.

## Introduction

### Background

Chronic diseases are the leading cause of death globally [1]. There are six key lifestyle risk behaviors linked to the development of chronic disease that often emerge during adolescence, highlighting adolescence as a potentially critical opportunity to intervene before these health behaviors become entrenched [2,3]. The key lifestyle behaviors are poor diet, physical inactivity, smoking, alcohol use [1,4,5], sedentary behavior (ie, sitting and recreational screen time) [6,7], and poor sleep (ie, long or short duration and poor quality) [8]. These *Big 6* risk behaviors have been found to commonly co-occur, which presents opportunities to adopt a multiple health behavior change approach [9]. Targeting multiple behavioral risk factors together, rather than in isolation, allows skills and knowledge learned in relation to one behavior to be transferred to other behaviors [10], resulting in improvements across multiple behaviors without additional effort [11]. To date, most chronic disease prevention and treatment approaches have been conducted among adults and have typically focused on changing single behaviors only, presenting a significant opportunity for the development of more effective and efficient prevention programs in adolescence [12].

Smartphones have become an integral part of everyday life, and many adolescents use mobile apps to monitor and improve their health [13,14]. In Australia, early adolescence and the transition from primary school to secondary school (which takes place when students are aged approximately 12 years) is a turning point for mobile phone ownership. Roughly 1 in 3 individuals aged 11 years, compared with three-fourth of those aged 13 years, own a mobile phone in Australia. This then increases to 91% for those aged 14 to 17 years, with 94% of those mobile phones being smartphones [15]. Mobile phone–based interventions have been shown to be effective in improving a range of risk factors associated with chronic diseases among adults, including physical inactivity, poor diet, sleep, overweight and obesity, alcohol use, smoking, and mental health problems [16-20], with similar evidence emerging among adolescents [21-26]. In addition, through mobile devices, individualized interventions can be provided inexpensively to a large number of people, including those who are geographically isolated, at a time and place when they are ready to engage [27]. Therefore, mobile phones may be an ideal platform for engaging adolescents with interventions to prevent key chronic disease risk behaviors. However, to the best of our knowledge, no mobile health (mHealth) tools that simultaneously target all of the Big 6 risk behaviors among adolescents currently exist [28].

Recently, we developed the Health4Life school-based program, a web-based program to concurrently address the Big 6 chronic disease risk factors among Australian secondary school students. The universal (ie, delivered to all students, regardless of risk) prevention program is based on the successful Climate Schools program [29-31], which uses interactive cartoon storylines about a group of teenagers and is based on the principles of social influence theory [32]. This includes providing accurate, relevant, and developmentally appropriate information; normative content; and resistance skills training. The Health4Life program adopts a multiple health behavior change approach and provides simultaneous education about the Big 6 via 6 web-based cartoon modules delivered in schools to students aged between 12 and 13 years (grade 7 in Australian secondary schools). More information about the development of this school-based program is given in a study by Champion et al [33]. Given that mobile phone–based interventions have previously demonstrated effectiveness in improving some of the Big 6 risk behaviors among adolescents [21-26,34], an accompanying smartphone app, the Health4Life app*,* was developed. The Health4Life app aims to extend the reach and reinforce the content of the school-based program as students progress from grades 7 to 9 and help engage and further encourage students to modify their behaviors via goal setting and self-monitoring—2 key evidence-based behavior change techniques (BCTs) [35-39].

The Health4Life app was based on our team’s previously developed and evaluated multiple health behavior change mobile-based tool named MyHealthPA [40]. MyHealthPA was developed to help people improve 4 of the Big 6 lifestyle risk behaviors (poor diet, physical inactivity, alcohol use, and smoking). MyHealthPA uses self-monitoring, goal setting, and provision of feedback via interactive progress graphs to help users improve their health behaviors. The design and delivery of MyHealthPA was informed by the Flat-Explicit Design Model [41], a model designed to reduce the cognitive effort required to effectively interact with digital health tools, increasing the ease of use among people with mental health problems. For example, the model recommends using a *flat design*, including no more than 2 levels past the initial page or screen, descriptive labels, and explicit instructions, rather than succinct and abstract labels, with text written at a low reading level. The MyHealthPA app was trialed among 28 young people aged between 19 and 25 years over an 8-week period [40]. Small improvements in fruit and vegetable consumption, level of physical activity, alcohol use, and mood were found between baseline, immediately postintervention, and 1 month follow-up [40]. However, several opportunities for improvement were also identified, including conversion to a native app format (as opposed to a responsive website optimized for mobile phones) [40].

The MyHealthPA tool provided the initial structure for the Health4Life app, and additional design elements were informed by the theoretical, design, and engagement framework of the Health4Life and Climate Schools programs [29,30,33,42]. The development was informed by the processes outlined later.

### Objectives

This paper aims to describe the co-design process and usability and acceptability testing of the Health4Life app.

## Methods

### Co-design

To assist with the conversion of the MyHealthPA tool to an app appropriate for adolescents that also complemented the Health4Life school-based program, we undertook an iterative development process. End users (adolescents) and experts (academics and clinicians with expertise in prevention, substance use, physical activity, exercise physiology, sleep, dietetics, mental health, eHealth interventions, and behavior change) were engaged repeatedly at key points in the process. The development process consisted of three key stages: stage 1, scoping of the literature; stage 2, end user consultations; and stage 3, app development and beta testing. Following this development work, the Health4Life app was included as an intervention component in the Health4Life trial (a large cluster randomized trial currently underway [43]), and we collected and analyzed information regarding the app’s usability and acceptability among 232 initial users.

#### Stage 1: Scoping of the Literature

We conducted a series of nonsystematic, structured literature reviews to identify key BCTs to improve the Big 6 lifestyle risk behaviors among adolescents and successfully engage them with an mHealth app. The synthesized findings of each of these searches are briefly discussed later. Where possible, we drew findings from previously published systematic reviews.

##### Effective BCTs Among Adolescents

The key BCTs used within the MyHealthPA tool to improve users’ health behaviors are self-monitoring and goal setting. Strong evidence for the efficacy of these BCTs among adults, including within mobile apps, exists [35-39]. To explore whether this evidence extends to adolescent populations, we conducted literature searches in PubMed. PubMed searches used terms, including self-monitoring, goal setting, health behavior, and adolescent or young people. We then explored whether evidence for other key BCTs among adolescents exists via a literature search using the following terms: BCTs and adolescent or young people.

Systematic reviews conducted by Rose et al [44], Brannon et al [34], and Willmott et al [45] found both self-monitoring and goal setting to be common components in behavior change interventions designed for adolescents. In a review of digital interventions to improve diet quality and physical activity among adolescents by Rose et al [44], they identified 11 studies that used goal setting and 14 that used self-monitoring and, of these, 6 used both strategies to encourage behavior change. Almost all the interventions that included goal setting showed significant improvements in adolescents’ diets, physical activity, or both. Their findings also suggested that self-monitoring and goal setting are most effective when paired. Similarly, in their systematic review of the pediatric literature on BCTs, Brannon et al [34] found that self-monitoring and BCTs related to goal setting significantly predicted improvements in physical activity for adolescents.

Although goal setting has been shown to be an effective technique for behavior change in adults, and increasingly in adolescents, concerns remain regarding its feasibility and effectiveness among adolescents [46]. Setting an appropriate goal requires skills such as abstract reasoning, which only begins to develop during adolescence. One solution is to preset goals for adolescents participating in behavior change interventions. Although there is evidence that goal-setting interventions can significantly improve physical activity behavior, regardless of who prescribes the goal [47], it has been suggested that presetting adolescents’ goals may limit their autonomy and decrease their commitment to that goal [46,48]. Instead, Shilts et al [46] suggested adopting a guided goal-setting approach in which adolescents select a broad goal area they would like to work on from a predefined list and are then presented with several minor related goals from which they can choose. This type of goal setting ensures the selection of appropriate goals in a way that empowers and respects adolescents’ autonomy. Testing of this approach suggests that it is an effective method, with between 87% and 89% of adolescents who set eating or physical activity goals in this way, reporting that they made an effort to reach their goals [46].

Our review of the literature identified several BCTs (beyond goal setting and self-monitoring) that are commonly used in behavior change interventions (including mobile-based ones) designed for adolescents [19,49,50], including social support [34,51], prompts or cues [52-55], modeling [34], providing consequences for behavior [34,51], and feedback on behavior [51]. However, few studies have investigated their efficacy in actually changing health behaviors among adolescents.

##### Designing an Engaging Health App for Adolescents

A literature search in PubMed was conducted to identify the best way to encourage adolescents to engage with health apps. The search included the terms mobile app, smartphone, mobile phone, mHealth, digital, engagement, adherence, and adolescent or young people.

A total of 2 recent systematic reviews have investigated the efficacy of different strategies and features used to engage adolescents in digital health interventions [19,56]. Partridge and Redfern [56] found that key strategies for effective engagement included co-designing with adolescents, personalization or tailoring of interventions, and just-in-time adaptation allowing the provision of personalized support based on an individual’s current context. A systematic review by Schoeppe et al [19] found that higher quality apps, in terms of functionality and user ratings, included more app features (such as educational information, social networking options, and gamification) and BCTs. However, this study also highlighted that more research is needed to discover which BCTs have a greater effect on engagement among this population.

Similarly, a recent review by Torous et al [57] identified five potential factors that contribute to low user engagement with mental health apps: (1) poor usability because of excessive burden of entering data, (2) lack of user-centric design because of poor involvement from the targeted population in development, (3) concerns regarding privacy, (4) a lack of trust because of unsubstantiated claims and lack of evidence, and (5) lack of easily accessible crisis support information.

Other potentially useful strategies for encouraging greater engagement with health apps among adolescents include providing awards or badges for completing app tasks and *streaks* (ie, completion of app tasks a number of days in a row) [58,59], providing funny or inspirational quotes or memes [60], and providing trigger or prompts to access the app. Many of these techniques have already been used in commercial apps to encourage engagement [57,61]. Although the actual efficacy of these strategies among adolescents is yet to be systematically tested, there are strong theoretical justifications for why their inclusion could improve adolescents’ engagement with a health app.

##### Key Implications for App Development

Taken together, our scoping of the literature suggests that self-monitoring and goal setting (employing a guided goal-setting approach) are appropriate and effective BCTs for use among adolescents. Of a number of other potentially appropriate BCTs identified in a small body of literature [34,51-55], providing prompts and cues and feedback on behavior are likely the best suited for inclusion in a mobile-based tool such as the Health4Life app. This study also reinforces the appropriateness of employing an approach that uses peers to model health behavior changes [33,34,62].

The importance of involving adolescents in the development of any health app has emerged as a key consideration for promoting engagement with mHealth apps [56,57]. Similarly, this study suggests that allowing individual customization of apps and personalization of feedback [56], ensuring that the app is easy to use [57], providing prompts or reminders [52-55], providing rewards [19], providing inspirational quotes [60], employing multiple engagement and behavior change strategies [19], providing easy access to additional support [57], being evidence based [57], and providing transparency regarding privacy issues [57], all may be important for improving engagement with health apps among adolescents.

#### Stage 2: End User Consultations

To understand mobile phone usage among the target age groups and inform the development of a prototype design, a web-based survey was conducted. A focus group was then conducted to gain more in-depth information and feedback on the prototype design to ensure that the Health4Life app was appropriate and tailored to the needs of adolescents.

##### Web-Based Survey With Adolescents

###### Participants and Procedure

A total of 7 independent secondary schools in metropolitan regions of New South Wales and the Australian Capital Territory, Australia, were invited to participate in an anonymous web-based survey. Of the 7 schools, 3 (2 coeducational and 1 female-only school) agreed to participate. Schools distributed information and consent forms to the parents or guardians of their students of grades 7 to 9. Opt-out written or verbal parental consent and active written student consent were required (815/816, 99.9% consent rate). The students completed the survey during classes between August and September 2018. Participants were entered into the draw to win a Fitbit valued at Aus $450 (US $315). Ethics approval was obtained from the University of New South Wales Sydney Human Research Ethics Committee (HC180224).

###### Measures

The web-based survey assessed the demographic characteristics, including age, sex, and postcode, and the Big 6 health behaviors and included a series of bespoke and adapted items [63] to assess mobile phone use. Participants were asked if they owned a smartphone, when and how often they used their smartphone, and how often they used their phone to undertake different activities and to describe their school’s policy regarding mobile phone use. Students were also asked to name their favorite health app and describe what they liked most about the app, including what motivated them to start using it and what features of the app they found most useful or helpful.

###### Analysis

Descriptive analyses were conducted using IBM SPSS Statistics 24 (IBM Corporation) to investigate sample characteristics and prevalence rates of mobile phone use. For open-ended responses collected, the sample was stratified by age and year group and a random subsample between 20% (163/815) and 25% (204/815) was selected to ensure balanced representation across age and year groups. These responses were qualitatively analyzed until data saturation was reached. Using an inductive approach [64], one author (LT) coded the responses and grouped them according to key themes.

###### Findings

A total of 815 students, including 687 (84.3%) females, 110 (13.5%) males, and 12 (1.5%) students who were identified as nonbinary or preferred not to disclose their gender, participated in the web-based survey. They were aged between 12 and 15.75 years (mean 13.89, SD 0.89 years). The Index of Community Socio-Educational Advantage (ICSEA) values for participating schools ranged between 1106 and 1182 [65]. The ICSEA values were calculated on a scale with a median of 1000 and SD of 100, with higher values indicating higher levels of educational advantage. Most students (725/815, 88.9%) owned a smartphone, whereas 6% (49/815) did not own a smartphone or were not sure if their phone was a smartphone. Table 1 displays the patterns of smartphone use reported by participants. Participants reported high frequencies of smartphone use overall but infrequent use during school hours. Table 2 shows the patterns of participants’ smartphone use for a variety of activities. The most frequently performed activities included accessing the internet, using apps, and sending or receiving text messages.

When asked to list and describe their favorite health app on their smartphone, 472 participants listed 84 different apps as their *favorite health app*. The most frequently mentioned app was the iPhone *Health* app, which is one of the preinstalled apps on the iPhone, with 22.7% (107/472) of students identifying it as their favorite health app. This was followed by 15% (71/472) of participants who identified the Fitbit app as their favorite, 9.5% (45/472) who listed Clue or Flo (menstrual cycle tracking apps), and 5.3% (25/472) who listed MyFitnessPal.

The key themes that emerged from the open-ended responses are listed in Table 3. When asked to describe their motivations for downloading and using these apps, participants primarily described a desire for knowledge or a desire to improve health and health behaviors that had motivated them to download the app. When asked about the features of their favorite app, participants described tracking features and providing guidance on health behaviors and issues to be the features they found most useful and helpful. However, a number of participants were unable to articulate the most useful features of their favorite health apps.

**Table 1:** Patterns of smartphone use among web-based survey participants who own a smartphone (N=815).

Patterns of smartphone use		Value, n (%)
**Frequency of smartphone use (n=725)**		
	Several times an hour	104 (14.3)
	Every hour	50 (6.9)
	Several times a day	326 (45.0)
	Every day	187 (25.8)
	Several times a week	41 (5.7)
	Once a week	14 (1.9)
	Monthly or less	1 (0.1)
	I do not use a smartphone	2 (0.3)
Use a smartphone during recess (n=725)		131 (18.1)
Use a smartphone during class (n=725)		45 (6.2)
Use a smartphone in between class (n=725)		77 (10.6)
Only use a smartphone on the way to or from school (n=725)		496 (68.4)
Do not use a smartphone during school hours (n=725)		166 (22.9)
**Carry a smartphone during a school day (eg, in a pocket or bag; n=721)**		
	Always	205 (28.4)
	Usually	127 (17.6)
	About half the time	74 (10.3)
	Rarely	142 (19.7)
	Never	173 (24.0)
School permits mobile phone use by students at school (n=721)		62 (8.6)

**Table 2:** Frequency of smartphone use for specific activities (n=719).

Smartphone use for specific activities	Frequency of smartphone use for activities, n (%)				
	Daily	Weekly	Once or twice a month	Less than once a month	Never
Make or receive phone calls	339 (47.1)	275 (38.2)	63 (8.8)	31 (4.3)	11 (1.5)
Send or receive text messages	568 (79.0)	108 (15.0)	28 (3.9)	9 (1.3)	6 (0.8)
Access the internet	594 (82.6)	76 (10.6)	24 (3.3)	13 (1.8)	12 (1.7)
Use apps	589 (81.9)	81 (11.3)	27 (3.8)	10 (1.4)	12 (1.7)
Social networking (eg, Facebook or Twitter)	533 (74.1)	68 (9.5)	18 (2.5)	7 (1.0)	93 (12.9)
Send or receive email	234 (32.6)	209 (29.1)	116 (16.1)	68 (9.5)	92 (12.8)
Take a picture	304 (42.3)	293 (40.8)	78 (10.8)	32 (4.5)	12 (1.7)
Look for health or medical information or track your health and fitness	102 (14.2)	150 (20.9)	168 (23.4)	121 (16.8)	178 (24.8)
Entertainment (listen to music and watch videos)	519 (72.2)	131 (18.2)	28 (3.9)	24 (3.3)	17 (2.4)

**Table 3:** Summary of key themes extracted from students’ open-ended responses.

Theme				Example
**Motivations for downloading and using health apps**				
	**A desire for knowledge**			
		Desire to better understand menstrual cycle (female respondents)	“My doctors told me to track my period and I kept forgetting to write it on paper, but I saw a you-tuber use an app to track their period and decided to give it a go.” (Female, aged 12 years)	
		Desire to better understand one’s own health, health behaviors, and mental health, often to help change behaviors	“MyFitnessPal provides every aspect from eating and weight health to physical health. I also needed an app which provided all of it so I had one place to store my health and progress.” (Male, aged 13 years) “What motivated me to download this app and then use it was that I wanted to keep a record of my health level, and later to see if the level increased or decreased and why. This also motivated me to increase my health level and be involved with more sporting activities.” (Female, aged 12 years)	
	**Desire to improve health and health behaviors**			
		Wanting to improve fitness, lose weight, get healthy, and improve sleep	“Wanted to get more fit.” (Female, aged 12 years) “Because I am very unactive and unfit.” (Female, aged 12 years)	
	Other motivations		To be more organized (eg, with health appointments) They only downloaded the app to assist a wearable device to function (eg, Fitbit app) Others had recommended they download the app and use it The app came preinstalled on their phone	
**Useful features of favorite health apps**				
	**Tracking**			
		Tracking a wide range of health issues and behaviors perceived to be an attractive feature of health apps (eg, tracking steps; sleep; exercise sessions; the distance and speed of runs and walks; menstrual cycles; and food intake, including detailed calorie counting and mood)	“It counts my exercise, my activity and my steps.” (Male, aged 14 years) “You can enter what you eat in a day and it will tell you if you need to stop eating something or eat something.” (Female, aged 13 years) “Allows me to track my feelings and health.” (Female, aged 14 years)	
	**Providing guidance**			
		Most useful features of health apps described to be features that provided guidance on health issues and behaviors (eg, guidance on workout routines; healthy foods, including recipes and scanning food items; and how to improve mood)	“The healthy recipes and the way I can scan food items to find out if they are healthy or not.” (Female, aged 13 years) “The recommended workouts for my age.” (Male, aged 12 years) “It has designed workouts that you can do and it shows you how to do them.” (Female, aged 13 years)	
	Other useful features		Entertainment Goal setting Reminders to perform healthy behaviors Allowing communication or competition with friends	

###### Key Implications for App Development

The findings from the web-based survey found that almost all adolescent smartphone owners reported using their smartphones at least daily (667/725, 92%). Most also reported using mobile apps on a daily basis (589/725, 81.9%). However, only 35.1% (252/725) reported daily or weekly use of their smartphones for health purposes. Together, these results support the use of a smartphone app to engage adolescents; however, they highlight the need for co-design and end user feedback when developing health apps to maximize engagement and potential health benefits.

Students reported sending or receiving text messages and accessing social networking sites far more frequently than making or receiving phone calls. This suggests that text messages are a key form of communication for this group and highlights that using text-based approaches (eg, apps and SMS) to deliver remote interventions is likely to be more familiar and potentially more acceptable to adolescents than telephone call–based approaches.

A desire to better understand their own health behaviors, often to help improve health behaviors, has emerged as a key motivation for using health apps. In designing a health app for this population, providing ways for adolescents to receive personalized feedback about their own patterns of behaviors may be important. In line with this, the ability to track health behaviors in an app has emerged as a desirable feature.

Finally, participants reported that very few of their schools permitted mobile phone use by students at school. The majority reported only using their smartphones on the way to or from school, although most did carry their phones with them during the school day. These insights have important implications for the design of apps and notification schedules for school-age adolescents. Although designing an app to collect ecological momentary assessments of health behaviors multiple times throughout the day may be a way to elicit a more accurate recording of health behaviors, these results suggest that such an approach would be unacceptable (encouraging students to break their schools’ mobile phone policies) and not feasible for school-age students. Prompting data entry before or after school may be a more acceptable approach.

##### Focus Group

A focus group with adolescents was conducted to gain feedback and suggestions for app content and design.

###### Participants and Procedure

Participants were 12 adolescents recruited via personal networks. Active written consent was obtained from parents and participants before their participation in the focus group. Participants attended a 1.5-hour long face-to-face focus group in January 2019, which was loosely directed by one researcher (LT) with open-ended and prompting questions.

Participants were shown an example of how the app may display tracked health behaviors using a prototype design, before being asked to comment on the example. Participants were then asked to make suggestions for alternative ways to show this information, including by drawing their ideas. Participants were also asked for their thoughts and suggestions regarding how the goal-setting section of the app could function and be presented. The focus groups were audio recorded and transcribed. The participants received a JB-HiFi gift voucher of Aus $20 (US $15). Ethical approval was obtained from the University of New South Wales Sydney Human Research Ethics Committee (HC17852).

###### Analysis

The focus group data were transcribed by one researcher (BO). Using an inductive approach, comments and recommendations were grouped into themes by one researcher (BO), cross-checked by a second researcher (LT) and then used to inform refinements and modifications.

###### Findings

A total of 12 female adolescents aged between 11.1 and 14.9 years participated in the focus group. All participants lived in Sydney, Australia. Participants commented that all data entry features and progress graphs within the app should be clear and easy to use. They mostly agreed that tracked health behaviors should be individually displayed across a week using line graphs (as opposed to displaying all 6 behaviors of interest on a single graph) and that progress graphs should also contain detailed summary information about health behavior. Examples of ways in which participants suggested health behavior tracking could be displayed within the app are available in Multimedia Appendix 1.

Most participants emphasized the importance of making goal setting and achievement a rewarding experience, with the inclusion of *winning* an icon, badge, or emoji when reaching a goal. Many participants also preferred the inclusion of motivational comments or explanations as to why one should attempt to reach each health behavior goal. Participants discussed the idea of *competing* with friends by sharing their behavior tracking with others; however, the group disagreed about whether this would be beneficial or detrimental for adolescent users. Other comments included ensuring that goals are easy to input and that the goals suggested by the app are achievable and easy to fit into pre-existing schedules.

###### Key Implications for App Development

Beyond design ideas for the display of progress graphs within the app, key takeaway messages from the focus group included the need to display progress for each of the 6 health behaviors of interest separately and provide a detailed summary of user behaviors. The need for a simple-to-use interface and the importance of providing rewards within the app to engage adolescents were also reiterated.

#### Stage 3: App Development and Beta Testing

The findings from stages 1 and 2 were used to inform the development of the Health4Life app. Using the MyHealthPA tool for initial structure and content and incorporating key recommendations from the formative research described earlier, an external IT development company was engaged to produce a beta version of the Health4Life app. Graphic designs developed for the Health4Life school-based program (eg, cartoon characters, icons, or fonts) were used to ensure consistency between the 2 complementary programs.

Initial usability testing of the beta version of the app was first undertaken, and any technical issues identified were resolved before the app was reviewed. The reviewers consisted of academics and clinicians with expertise in adolescent health behavior change (n=6) and 4 adolescents within the teams’ personal networks. Two of these adolescents had also participated in the focus group. The 2 other adolescents were males who were unable to participate in the focus group; however, the research team felt it was important to obtain the input of male adolescents. Reviewers provided feedback regarding the final content, usability, acceptability, and appeal of the program.

Key changes to the beta version suggested by reviewers included improvements in the way in which content was displayed within the app (eg, simplifying health behavior progress graphs, adding stars on the dashboard to indicate when users reported meeting recommended health behavior guidelines to help users easily track progress for the day and reinforce healthy behaviors) and minor content changes (eg, removing quotes from celebrities perceived not to be current enough to be of interest to end users and adding quotes from current Australian-based celebrities).

### The Final Health4Life App

**Figure 1:**
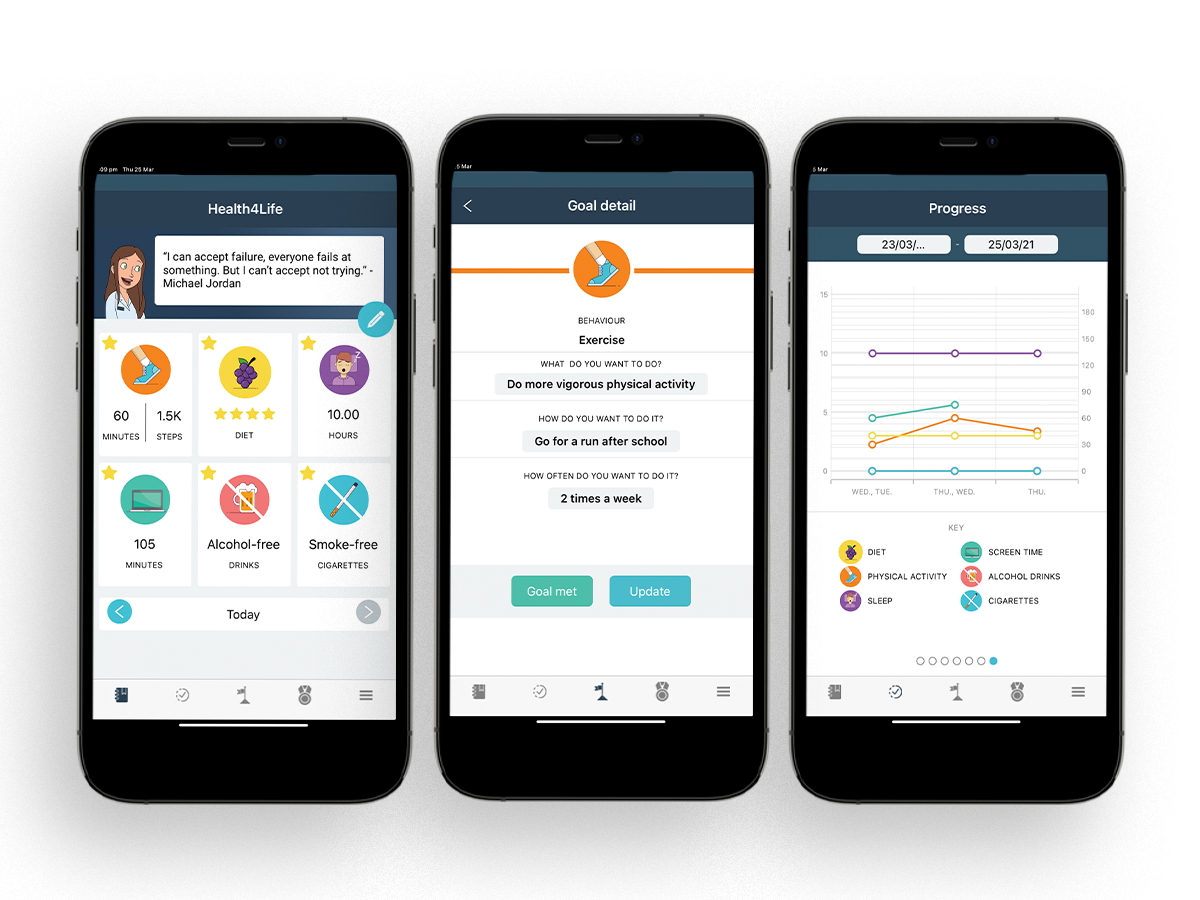
Selected screenshots of the Health4Life app.

The resulting Health4Life app consisted of the following 6 sections (screenshots are shown in Figure 1).

#### Dashboard

This page provides a simple and visual portrayal of the user’s Big 6 health behaviors for the current day and a menu to access all other pages. When users report meeting the recommended guidelines for a particular behavior, a gold star appears next to the relevant health behavior. To promote peer-to-peer communication and education, users are prompted to select a *Health4Life Buddy* to help guide them through the app from the group of 6 core characters in the school-based program. On the dashboard their *buddy* presents different motivational celebrity quotes, tips, and prompts to log behaviors.

#### Diary

This page allows users to record their health behaviors and mood. For each behavior, users can click an icon to give them more information about what exactly they are being asked to enter (eg, what a serve of fruit is or a definition of recreational screen time). Textbox 1 describes the specific behaviors that the user can record.

**Daily behaviors recorded within the Health4Life app.**

Lifestyle risk behavior and specific behaviors recorded

- Physical activity
  Minutes of moderate-to-vigorous physical activity
  Steps (manual entry)
- Diet
  Number of sugar-sweetened beverages
  Serves of fruit
  Serves of vegetables
  Number of discretionary food items
- Sleep
  Bedtime (the previous night)
  Wake-up time (that morning)
  Recreational screen time
  Minutes of recreational screen time
- Alcohol use
  If they have drunk any alcohol (yes or no)
  Number of standard drinks of alcohol (including just a sip)
- Tobacco use
  If they have smoked any tobacco (yes or no)
  Number of cigarettes smoked (including just a puff)
- Mood
  How they felt (5-point emoticon scale)

#### Progress

This section allows users to view their progress individually for each of the 6 health behaviors. Users are also provided with a summary of their behaviors, which highlights for how many days in the previous 7 days they have met the recommended guidelines for that behavior. A final progress graph displays all 6 behaviors together, allowing users to identify ways in which their health behaviors may be linked.

#### Goals

This section allows users to complete a guided goal-setting activity to set specific, measurable, achievable, relevant, time-bound (SMART) goals for any of the Big 6 behaviors. Users are prompted to first select the behavior they would like to set a goal for. They are then presented with 2 to 4 broad goal options: *What they would like to do* (eg, do more light physical activity, do more moderate physical activity). Upon selecting their broad goal, they were asked, “How would you like to do it?” and presented with up to 7 appropriate options for how they might achieve that goal (eg, go for a jog after school, dance to music, play a game like soccer, handball, or tag during lunch or recess at school) and prompted to select *How often they would like to do it?* (once a week, 2 times a week, 3 times a week, etc).

#### Rewards

This section allows users to view how many medals they have received and access information about what they need to do to earn medals for each health behavior. For each behavior, users can earn a bronze medal for meeting recommended guidelines for that behavior for 1 day, a silver medal for meeting the recommended guidelines for 3 days in a row, and a gold medal for meeting the recommended guidelines for that behavior for 7 days in a row.

#### Menu

In this section, users can access their profile and app settings along with the following:

- Resources: links and a brief description of web-based resources users could access for extra information and additional support to change their health behaviors and improve their mental health.
- Emergency: contact details for relevant chat and telephone help lines. Users are instructed to contact one of these services or contact emergency services if they are thinking about suicide or experiencing a personal crisis.
- Help: link to a video tutorial on how to use the Health4Life app and details of how to get help with any technical issues using the app.

### Usability and Acceptability Testing

Following the development work described earlier, access to the Health4Life app was provided to 3623 intervention condition students participating in the Health4Life trial. Students were provided with details on how to access the app via email (ie, downloading from the relevant app store), and teachers were asked to encourage students to download the app. After logging on to the app, students were sent daily reminder messages each evening, prompting them to record their health behaviors in the app. The Health4Life trial is a cluster randomized controlled trial currently being conducted among 6640 students from 71 schools across New South Wales, Western Australia, and Queensland, Australia (trial commenced in 2019; full trial details are given in the study by Teesson and Champion [43]).

Students at independent secondary schools in New South Wales that were allocated to the intervention condition in the Health4Life trial (7 schools in total; ICSEA values ranging between 1012 and 1139 [65]) were invited to complete a web-based questionnaire evaluating the Health4Life school-based program and the Health4Life app. Items related to the app aimed to generate information regarding the usability and acceptability of the app and included the System Usability Scale (SUS) [66], a valid and reliable industry standard tool for measuring the usability of digital tools [67]. Items were measured on a 5-point scale ranging from strongly disagree to strongly agree. Participants were also asked to name 1 good thing and 1 bad thing about the app or to give a reason for not downloading the app. These open-ended responses were coded and grouped into themes by one author (BO) until saturation was achieved.

## Results

A total of 535 students (535/685, 78.1%) completed the questionnaire; of these, 232 (43.4%) reported downloading the app. Overall, students rated the Health4Life app favorably, with 58.2% (135/232) rating the app as either good or very good and only 6.9% (16/232) rating the app as poor or very poor. Approximately half of the participants (127/232, 55%) agreed or strongly agreed that the app would help people of their age to change their lifestyle behaviors (17/232, 7.3% disagree or strongly disagree), and 42.4% (98/232) reported that they would recommend the app to their friends (42/232, 18.1% maybe; 91/232, 39.2% no).

Responses to the SUS found that most students thought the Health4Life app was easy to use (124/232, 53.4%), that they would not need the help of a technical person to be able to use the app (135/232, 58.4%), that most people would learn to use the app very quickly (127/232, 54.7%), that they felt very confident using the app (122/232, 52.5%), that the app was not unnecessarily complex (106/232, 45.7%), and that they did not need to learn a lot before they could get going with the app (106/232, 45.7%).

Some participants reported that the app was cumbersome to use (41/232, 17.6%), there was too much inconsistency within the app (33/232, 14.2%), or the features of the app were not well integrated (24/232, 10.3%). However, most responses were neutral for these items (143/232, 61.6%; 109/232, 46.9%; 126/232, 54.2%, respectively). Similarly, only 24.1% (56/232) of students reported agreeing or strongly agreeing that they would like to use the app frequently. The app received a total average SUS score of 59.5 (SD 14.1), representing *OK* usability [68].

Positive things about the app design identified by students included its esthetic design (eg, “It looks cool”), general structure of the app, and that it was easy to use (eg, “It was easy to understand”). However, negative aspects of the app’s design highlighted by students included its simplistic design and lack of advanced features (eg, “I found it a little bit basic for a fitness app, as I thought it would have more features”) and the design of the progress graphs (eg, “I would have liked my data and rating on my health to be displayed differently so it is easier to read”).

App features that students highlighted as positive included the mobile format (eg, “It is useful to use because it is on your phone or iPad”) and the ability to track multiple health behaviors (eg, “It tracks all the different parts to make sure you have a healthy lifestyle”), set goals, and the reminders sent (eg, “It gave me helpful notifications to use the app”). The ability to personalize the app and receive individualized feedback about health behaviors was also very well received (eg, “The app helps YOU discover YOUR habits and how to change them, with a supportive character. The overall app is very personalised”).

A common complaint from participants regarding the app’s features was the need to enter steps manually into the app, rather than being able to link to a step counter device (eg, “It could be connected to your phone…and it would count your steps and exercise”).

Participants also discussed that remembering their health behaviors over the course of the entire day can be difficult. Although some participants found reminders useful, others expressed that the reminders to use the app were annoying (eg, “it notifies me all the time and it is annoying”). From a technical side of things some participants also complained about the app sometimes freezing or not working properly (eg, “it can sometimes be a bit glitchy and/or freeze”).

Finally, among those participants who did not download the app, the top reasons for not doing so were not having a phone or enough phone storage, feeling like they did not need the app or the Health4Life school-based program was enough, not knowing that there was an app associated with the Health4Life school-based program, or not being informed how to download it (including that they were told by their teacher not to download the app or that they did not have to download it).

## Discussion

### Principal Findings

This study aims to describe formative research that led to the development of the Health4Life app and the initial usability and acceptability testing. Scoping of the literature identified key evidence-based BCTs, such as self-monitoring, goal setting, and providing prompts and feedback on behavior that were incorporated into the app design. End user consultations revealed that most adolescents (725/815, 88.9%) owned a smartphone that they frequently used to access the internet, use apps for social networking, and send or receive text messages; however, health apps were less frequently used, highlighting the need for co-design to increase engagement. Key app features, such as progress graphs and rewards, were identified and incorporated into the app design. Together, these findings led to the development of the Health4Life app, a co-designed, self-monitoring smartphone app, for adolescents that concurrently targets the Big 6 lifestyle behaviors.

Initial acceptability and usability findings suggest that the Health4Life app is an easy-to-use tool with features that appeal to adolescents. Approximately half of the respondents included in our feasibility and acceptability testing rated the Health4Life app as *good* or *very good* and agreed that the app was easy to use and would help people like them to change their lifestyle behaviors. These positive perspectives were reiterated in many open-ended responses from the participants. However, only a quarter of participants reported that they would like to use the app frequently and most participants expressed neutral attitudes with regard to the app consistency and integration of features. Despite previous support for the effectiveness of mobile phone–based interventions for changing health risk behaviors [16-20], disparity between perceived acceptability of an app and actual use has been reported in previous mobile-based development and evaluation studies [40]. Co-design with young people and tools to promote active engagement are likely to be important components of any youth-based app.

### Strengths and Limitations

Formative research that informed the development of the Health4Life app had several limitations. First, the reviews of the literature were not formal systematic reviews. Other useful features to incorporate into the Health4Life app may have been identified if a more comprehensive review of the literature was conducted. However, the features that were included in the app, to encourage either health behavior change or engagement (eg, self-monitoring, goal setting, personalized feedback, or rewards), have a strong theoretical and empirical evidence base to support their use. Another limitation was that the end user consultations only included students from metropolitan areas and involved mainly female participants, with no males participating in the focus group. Different perspectives and ideas for app content presentation may have been generated if more males and students from nonmetropolitan areas had been recruited. The full trial of the Health4Life program is currently underway and will allow us to evaluate the acceptability and effectiveness of the Health4Life app in a broader sample of gender-diverse adolescents (N=6640) [43]. Furthermore, because of limited time and resources, coding and grouping of qualitative data was completed by only one researcher.

Although very few participants reported negative views of the usability of the app, it is clear that there is some room for improvement, given the less-than-optimal average SUS total score obtained (59.2, which reflects a *D grading* and *OK* usability). Results from the SUS are displayed in Multimedia Appendix 2. However, similar scores have been reported for other mHealth apps with comparable features (eg, goal setting, rewards, and diaries for health behaviors) when reviewed by adolescents in several European countries [69]. It is also worth noting that the SUS score means that receiving all neutral responses will result in a score of 50 and *fail* grading. Our score, in part, reflects a high proportion of neutral responses received. Although these neutral responses may represent genuine neutral perspectives, research has shown that participants may use a midpoint response when they do not understand an item or if the item is ambiguous or socially undesirable [70]. In addition, questions offering neutral midpoints decrease response reliability and measurement quality in children and adolescents [71-75]. As such, the SUS responses from our sample of those aged 11 to 14 years may need to be interpreted with caution.

This formative research generated important insights into the development and implementation of health apps for adolescents. Additional customization with regard to the timing and frequency of reminder notifications may be important when trying to strike the right balance between enough reminders to effectively engage users and too many so that users are put off and choose not to access or disengage with the app. Participants in our initial examination of the feasibility and acceptability of the Health4Life app received a single notification via email, alerting them to the app and how they could access it. However, anecdotally, it emerged that a large proportion of students did not frequently check their school email addresses, which they were required to use as their primary email contact in the trial. Teachers were encouraged to mention the app to students and encourage them to download it and were provided with an example classroom activity they could conduct using the app. However, implementation of these activities was at the teacher’s discretion and results from our feasibility and acceptability testing showed that many teachers did not mention the app to students or in some cases discouraged their students from downloading it. Alternative modes of communication (eg, SMS text messaging) or parental engagement may be needed to effectively reach most students outside of the school environment and working more closely with teachers to address any concerns they might have in encouraging their students to download and use an app like the Health4Life app.

Finally, the feasibility and acceptability of the app was tested only among a subsample of participants who were all from independent secondary schools in the metropolitan regions of New South Wales. The full trial of the Health4Life program will evaluate the effectiveness of the school-based program and smartphone app at targeting the Big 6 risk behaviors among this subsample and students from 2 other states, including independent, public, and Catholic schools in both metropolitan and regional locations. As such, it is one of the largest and most diverse samples of Australian adolescents. This will enable us to examine the ways in which users interact with the app, how different patterns of use or nonuse might influence health behavior change outcomes among users, and how this might differ for different types of users.

### Conclusions

The Health4Life app is the first mobile app intervention specifically designed with, and for, adolescents to concurrently address the Big 6 risk behaviors. Designed in collaboration with adolescents and experts and adopting a multiple health behavior change approach, it has the potential to efficiently and effectively modify important risk factors for chronic diseases among young people.

As part of the Health4Life initiative, the Health4Life app is provided in conjunction with the Health4Life school-based program to all grade 7 students, regardless of their engagement in the Big 6 risk behaviors. To our knowledge, this is the first time such an app will be used to support a school-based program to simultaneously target the Big 6 lifestyle risk behaviors [28]. By reinforcing key learning from the school-based program outside of the classroom and allowing students to receive regular and individualized feedback about their own health behaviors, the Health4Life app has the potential to efficiently and effectively modify important risk factors for chronic disease among young people.

The next important step is to establish the effectiveness of the Health4Life intervention, including the Health4Life app. A cluster randomized controlled trial is currently underway in 71 schools across Australia to evaluate whether Health4Life is more effective than health education as usual in delaying the uptake of alcohol and tobacco use, reducing sedentary recreational screen time, reducing the decline in moderate-to-vigorous physical activity, reducing the consumption of sugar-sweetened beverages, and improving sleep [43].

## Appendix

Multimedia Appendix 1 Example of focus group participants’ suggestions for displaying tracked behaviors in the app.

Multimedia Appendix 2 System usability scale results.

## Abbreviations

BCT: behavior change technique
ICSEA: Index of Community Socio-Educational Advantage
mHealth: mobile health
SUS: System Usability Scale

## References

[1] Australian Institute of Health and Welfare (AIHW) (2014). Australia's health 2014. The 14th biennial health report of the Australian Institute of Health and Welfare.

[2] Wilcox S (2015). Chronic diseases in Australia: blueprint for preventive action. Mitchell Institute and Australian Health Policy Collaboration.

[3] de la Haye K, D'Amico EJ, Miles JNV, Ewing B, Tucker JS (2014). Covariance among multiple health risk behaviors in adolescents. PLoS One.

[4] Ezzati M, Riboli E (2013). Behavioral and dietary risk factors for noncommunicable diseases. N Engl J Med.

[5] World Health Organization (2013). Global action plan for the prevention and control of noncommunicable diseases 2013-2020.

[6] Ding D, Rogers K, van der Ploeg Hidde, Stamatakis E, Bauman AE (2015). Traditional and emerging lifestyle risk behaviors and all-cause mortality in middle-aged and older adults: evidence from a large population-based Australian cohort. PLoS Med.

[7] Lynch BM, Owen N (2015). Too much sitting and chronic disease risk: steps to move the science forward. Ann Intern Med.

[8] Cappuccio FP, Cooper D, D'Elia L, Strazzullo P, Miller MA (2011). Sleep duration predicts cardiovascular outcomes: a systematic review and meta-analysis of prospective studies. Eur Heart J.

[9] Prochaska JJ, Spring B, Nigg CR (2008). Multiple health behavior change research: an introduction and overview. Prev Med.

[10] Fleig L, Lippke S, Pomp S, Schwarzer R (2011). Intervention effects of exercise self-regulation on physical exercise and eating fruits and vegetables: a longitudinal study in orthopedic and cardiac rehabilitation. Prev Med.

[11] Johnson SS, Paiva AL, Mauriello L, Prochaska JO, Redding C, Velicer WF (2014). Coaction in multiple behavior change interventions: consistency across multiple studies on weight management and obesity prevention. Health Psychol.

[12] Spring B, Moller AC, Coons MJ (2012). Multiple health behaviours: overview and implications. J Public Health (Oxf).

[13] Barratt MJ, Lenton S (2010). Beyond recruitment? Participatory online research with people who use drugs. Int J Internet Res Ethics.

[14] Australian Communications and Media Authority (2016). Aussie teens and kids online. Office of the Children's eSafety Commissioner.

[15] (2016). Australian mobile phone owners aged 14-17. Roy Morgan Research.

[16] Bort-Roig J, Gilson ND, Puig-Ribera A, Contreras RS, Trost SG (2014). Measuring and influencing physical activity with smartphone technology: a systematic review. Sports Med.

[17] Mateo GF, Granado-Font E, Ferré-Grau C, Montaña-Carreras X (2015). Mobile phone apps to promote weight loss and increase physical activity: a systematic review and meta-analysis. J Med Internet Res.

[18] Rathbone AL, Clarry L, Prescott J (2017). Assessing the efficacy of mobile health apps using the basic principles of cognitive behavioral therapy: systematic review. J Med Internet Res.

[19] Schoeppe S, Alley S, Van Lippevelde Wendy, Bray NA, Williams SL, Duncan MJ, Vandelanotte C (2016). Efficacy of interventions that use apps to improve diet, physical activity and sedentary behaviour: a systematic review. Int J Behav Nutr Phys Act.

[20] Shin JC, Kim J, Grigsby-Toussaint D (2017). Mobile phone interventions for sleep disorders and sleep quality: systematic review. JMIR Mhealth Uhealth.

[21] Schoeppe S, Alley S, Rebar AL, Hayman M, Bray NA, Van Lippevelde Wendy, Gnam J, Bachert P, Direito A, Vandelanotte C (2017). Apps to improve diet, physical activity and sedentary behaviour in children and adolescents: a review of quality, features and behaviour change techniques. Int J Behav Nutr Phys Act.

[22] Quante M, Khandpur N, Kontos EZ, Bakker JP, Owens JA, Redline S (2019). A qualitative assessment of the acceptability of smartphone applications for improving sleep behaviors in low-income and minority adolescents. Behav Sleep Med.

[23] Wang K, Varma DS, Prosperi M (2018). A systematic review of the effectiveness of mobile apps for monitoring and management of mental health symptoms or disorders. J Psychiatr Res.

[24] Smith JJ, Morgan PJ, Plotnikoff RC, Dally KA, Salmon J, Okely AD, Finn TL, Lubans DR (2014). Smart-phone obesity prevention trial for adolescent boys in low-income communities: the ATLAS RCT. Pediatrics.

[25] Quelly SB, Norris AE, DiPietro JL (2016). Impact of mobile apps to combat obesity in children and adolescents: a systematic literature review. J Spec Pediatr Nurs.

[26] Dute DJ, Bemelmans WJE, Breda J (2016). Using mobile apps to promote a healthy lifestyle among adolescents and students: a review of the theoretical basis and lessons learned. JMIR Mhealth Uhealth.

[27] Brouwer W, Kroeze W, Crutzen R, de Nooijer J, de Vries NK, Brug J, Oenema A (2011). Which intervention characteristics are related to more exposure to internet-delivered healthy lifestyle promotion interventions? A systematic review. J Med Internet Res.

[28] Champion KE, Newton NC, Spring B, Wafford QE, Parmenter BJ, Teesson M (2017). A systematic review of school-based eHealth interventions targeting alcohol use, smoking, physical inactivity, diet, sedentary behaviour and sleep among adolescents: a review protocol. Syst Rev.

[29] Newton NC, Teesson M, Vogl LE, Andrews G (2010). Internet-based prevention for alcohol and cannabis use: final results of the Climate Schools course. Addiction.

[30] Newton NC, Vogl LE, Teesson M, Andrews G (2009). CLIMATE Schools: alcohol module: cross-validation of a school-based prevention programme for alcohol misuse. Aust N Z J Psychiatry.

[31] Teesson M, Newton NC, Slade T, Carragher N, Barrett EL, Champion KE, Kelly EV, Nair NK, Stapinski LA, Conrod PJ (2017). Combined universal and selective prevention for adolescent alcohol use: a cluster randomized controlled trial. Psychol Med.

[32] Kelman HC (2016). Compliance, identification, and internalization three processes of attitude change. J Confl Resolut.

[33] Champion KE, Gardner LA, McGowan C, Chapman C, Thornton L, Parmenter B, McBride N, Lubans DR, McCann K, Spring B, Teesson M, Newton NC, Health4Life Team (2020). A web-based intervention to prevent multiple chronic disease risk factors among adolescents: co-design and user testing of the Health4Life school-based program. JMIR Form Res.

[34] Brannon EE, Cushing CC (2015). A systematic review: is there an app for that? Translational science of pediatric behavior change for physical activity and dietary interventions. J Pediatr Psychol.

[35] Ward MC, White DT, Druss BG (2015). A meta-review of lifestyle interventions for cardiovascular risk factors in the general medical population. J Clin Psychiatry.

[36] Norris SL, Engelgau MM, Narayan KM (2001). Effectiveness of self-management training in type 2 diabetes: a systematic review of randomized controlled trials. Diabetes Care.

[37] Burke LE, Wang J, Sevick MA (2011). Self-monitoring in weight loss: a systematic review of the literature. J Am Diet Assoc.

[38] Jenkins RJ, McAlaney J, McCambridge J (2010). Corrigendum to “Change over time in alcohol consumption in control groups in brief intervention studies: Systematic review and meta-regression study” [Drug Alcohol Depend. 100 (2009) 107–114]. Drug Alcohol Depend.

[39] Swendeman D, Ramanathan N, Baetscher L, Medich M, Scheffler A, Comulada W (2015). Smartphone self-monitoring to support self-management among people living with HIV: perceived benefits and theory of change from a mixed-methods randomized pilot study. J Acquir Immune Defic Syndr.

[40] Thornton L, Kay-Lambkin F, Tebbutt B, Hanstock TL, Baker AL (2018). A mobile phone-based healthy lifestyle monitoring tool for people with mental health problems (myhealthpa): development and pilot testing. JMIR Cardio.

[41] Rotondi AJ, Eack SM, Hanusa BH, Spring MB, Haas GL (2015). Critical design elements of e-health applications for users with severe mental illness: singular focus, simple architecture, prominent contents, explicit navigation, and inclusive hyperlinks. Schizophr Bull.

[42] Teesson M, Newton NC, Slade T, Chapman C, Birrell L, Mewton L, Mather M, Hides L, McBride N, Allsop S, Andrews G (2020). Combined prevention for substance use, depression, and anxiety in adolescence: a cluster-randomised controlled trial of a digital online intervention. Lancet Digit Health.

[43] Teesson M, Champion KE, Newton NC, Kay-Lambkin F, Chapman C, Thornton L, Slade T, Sunderland M, Mills K, Gardner LA, Parmenter B, Lubans DR, Hides L, McBride N, Allsop S, Spring BJ, Smout S, Osman B, Health4Life Team (2020). Study protocol of the Health4Life initiative: a cluster randomised controlled trial of an eHealth school-based program targeting multiple lifestyle risk behaviours among young Australians. BMJ Open.

[44] Rose T, Barker M, Jacob CM, Morrison L, Lawrence W, Strömmer S, Vogel C, Woods-Townsend K, Farrell D, Inskip H, Baird J (2017). A systematic review of digital interventions for improving the diet and physical activity behaviors of adolescents. J Adolesc Health.

[45] Willmott TJ, Pang B, Rundle-Thiele S, Badejo A (2019). Weight management in young adults: systematic review of electronic health intervention components and outcomes. J Med Internet Res.

[46] Shilts MK, Horowitz M, Townsend MS (2004). An innovative approach to goal setting for adolescents: guided goal setting. J Nutr Educ Behav.

[47] McEwan D, Harden SM, Zumbo BD, Sylvester BD, Kaulius M, Ruissen GR, Dowd AJ, Beauchamp MR (2016). The effectiveness of multi-component goal setting interventions for changing physical activity behaviour: a systematic review and meta-analysis. Health Psychol Rev.

[48] Shilts MK, Horowitz M, Townsend MS (2009). Guided goal setting: effectiveness in a dietary and physical activity intervention with low-income adolescents. Int J Adolesc Med Health.

[49] Hsu MSH, Rouf A, Allman-Farinelli M (2018). Effectiveness and behavioral mechanisms of social media interventions for positive nutrition behaviors in adolescents: a systematic review. J Adolesc Health.

[50] Sawyer A, Lewthwaite H, Gucciardi DF, Hill K, Jenkins S, Cavalheri V (2019). Behaviour change techniques to optimise participation in physical activity or exercise in adolescents and young adults with chronic cardiorespiratory conditions: a systematic review. Intern Med J.

[51] Ashton LM, Sharkey T, Whatnall MC, Williams RL, Bezzina A, Aguiar EJ, Collins CE, Hutchesson MJ (2019). Effectiveness of interventions and behaviour change techniques for improving dietary intake in young adults: a systematic review and meta-analysis of RCTs. Nutrients.

[52] Morrison LG, Hargood C, Pejovic V, Geraghty AWA, Lloyd S, Goodman N, Michaelides DT, Weston A, Musolesi M, Weal MJ, Yardley L (2018). Correction: the effect of timing and frequency of push notifications on usage of a smartphone-based stress management intervention: an exploratory trial. PLoS One.

[53] Freyne J, Yin J, Brindal E, Hendrie GA, Berkovsky S, Noakes M (2017). Push notifications in diet apps: influencing engagement times and tasks. Int J Hum-comput Int.

[54] Alkhaldi G, Hamilton FL, Lau R, Webster R, Michie S, Murray E (2016). The effectiveness of prompts to promote engagement with digital interventions: a systematic review. J Med Internet Res.

[55] Bidargaddi N, Pituch T, Maaieh H, Short C, Strecher V (2018). Predicting which type of push notification content motivates users to engage in a self-monitoring app. Prev Med Rep.

[56] Partridge SR, Redfern J (2018). Strategies to engage adolescents in digital health interventions for obesity prevention and management. Healthcare (Basel).

[57] Torous J, Nicholas J, Larsen ME, Firth J, Christensen H (2018). Clinical review of user engagement with mental health smartphone apps: evidence, theory and improvements. Evid Based Ment Health.

[58] Zichermann G, Cunningham C (2011). Gamification by design: implementing game mechanics in web and mobile apps.

[59] Krikelas J (1983). Information-seeking behavior: patterns and concepts. Drexel Library Q.

[60] Werbach K, Hunter D (2012). For the win: how game thinking can revolutionize your business.

[61] Rabbi M, Philyaw-Kotov M, Lee J, Mansour A, Dent L, Wang X, Cunningham R, Bonar E, Nahum-Shani I, Klasnja P, Walton M, Murphy S (2017). SARA: a mobile app to engage users in health data collection. Proc ACM Int Conf Ubiquitous Comput.

[62] Vogl LE, Teesson M, Newton NC, Andrews G (2012). Developing a school-based drug prevention program to overcome barriers to effective program implementation: The CLIMATE schools: alcohol module. Open J Prev Med.

[63] Smith A (2011). Americans and their cell phones. Pew Research Center.

[64] Thomas DR (2016). A general inductive approach for analyzing qualitative evaluation data. Am J Eval.

[65] ACARA (2013). Guide to understanding 2013 Index of Community Socio-educational Advantage (ICSEA) values. Australian Curriculum Assessment and Reporting Authority.

[66] Brooke J (1996). SUS-A quick and dirty usability scale.

[67] Peres SC, Pham T, Phillips R (2013). Validation of the System Usability Scale (SUS): SUS in the wild. Proceedings of the Human Factors and Ergonomics Society Annual Meeting.

[68] Sauro J A practical guide to the systems usibility scale. MeasuringU.

[69] Martin A, Caon M, Adorni F, Andreoni G, Ascolese A, Atkinson S, Bul K, Carrion C, Castell C, Ciociola V, Condon L, Espallargues M, Hanley J, Jesuthasan N, Lafortuna CL, Lang A, Prinelli F, Puidomenech Puig E, Tabozzi SA, McKinstry B (2020). A mobile phone intervention to improve obesity-related health behaviors of adolescents across Europe: iterative co-design and feasibility study. JMIR Mhealth Uhealth.

[70] Kulas JT, Stachowski AA (2009). Middle category endorsement in odd-numbered Likert response scales: associated item characteristics, cognitive demands, and preferred meanings. J Res Pers.

[71] Alwin DF (2007). Margins of error: a study of reliability in survey measurement.

[72] Borgers N, de Leeuw E, Hox J (2016). Children as respondents in survey research: cognitive development and response quality 1. Bull Methodol Sociol.

[73] Borgers N, Hox J, Sikkel D (2004). Response effects in surveys on children and adolescents: the effect of number of response options, negative wording, and neutral mid-point. Quality & Quantity.

[74] Krosnick JA, Fabrigar LR (1997). Designing rating scales for effective measurement in surveys.

[75] Vaillancourt PM (1973). Stability of children's survey responses. Publ Opin Q.

