# Supplementary material for: A Multiple Health Behavior Change, Self-Monitoring Mobile App for Adolescents: Development and Usability Study of the Health4Life App
Source: JMIR Form Res. 2021 Apr 12;5(4):e25513. doi: 10.2196/25513 (PMC8076990; doi:10.2196/25513)
Supplement: Multimedia Appendix 1 [file formative_v5i4e25513_app1.docx]

# Multimedia Appendix 1

Example of focus group participant’s suggestions for displaying tracked behaviours in app


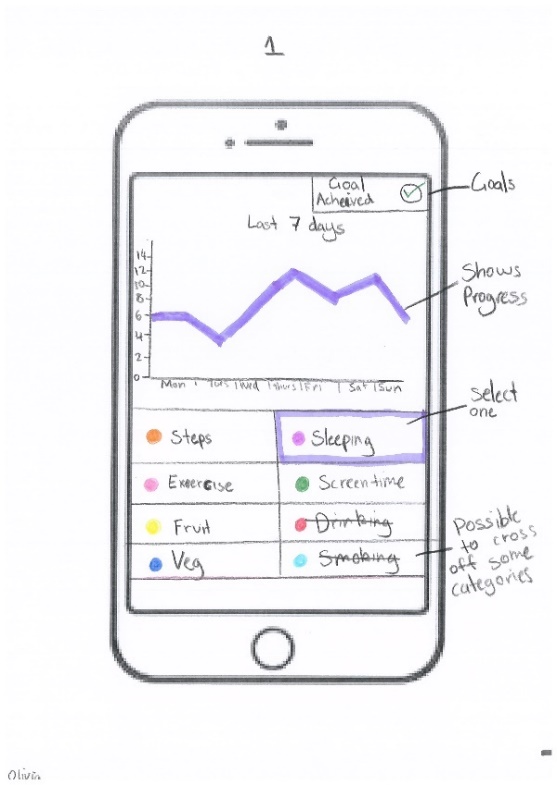

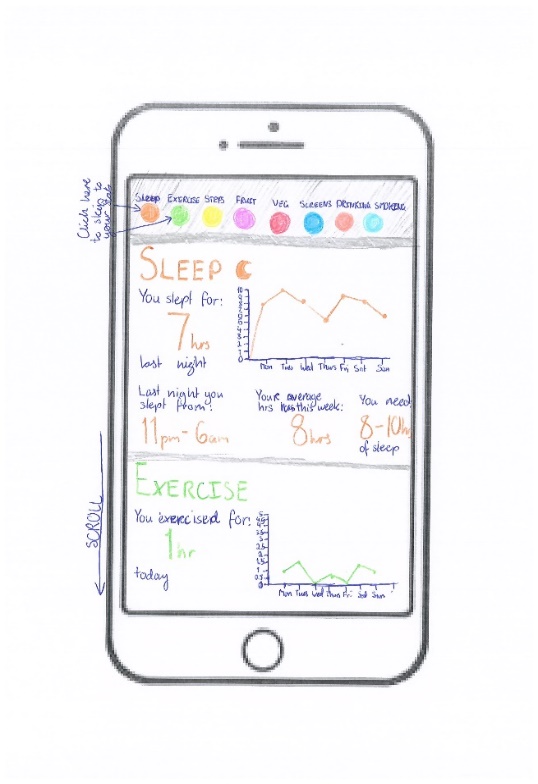

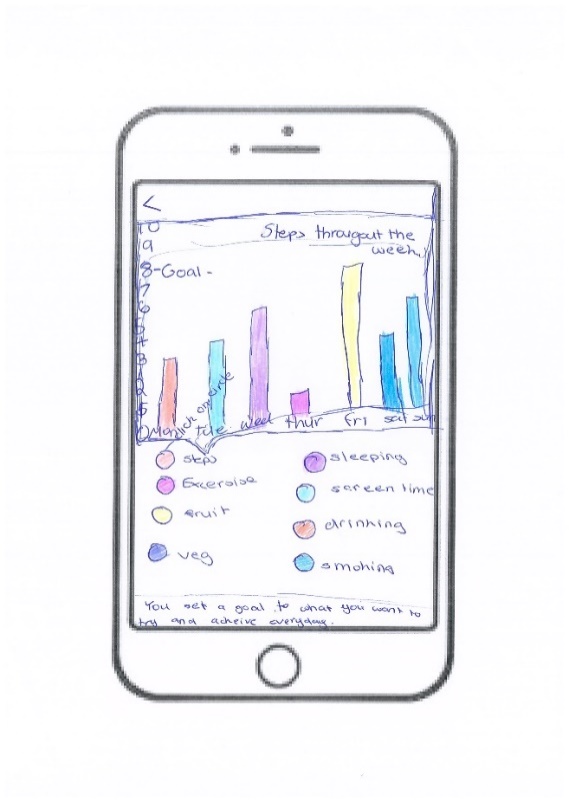


This is a Multimedia Appendix to a full manuscript published in the J Med Internet Res. For full copyright and citation information see http://dx.doi.org/10.2196/25513
