# Supplementary material for: A Multiple Health Behavior Change, Self-Monitoring Mobile App for Adolescents: Development and Usability Study of the Health4Life App
Source: JMIR Form Res. 2021 Apr 12;5(4):e25513. doi: 10.2196/25513 (PMC8076990; doi:10.2196/25513)
Supplement: Multimedia Appendix 2 [file formative_v5i4e25513_app2.docx]

# Multimedia Appendix 2

System usability scale results for the Health4Life app

| **System usability scale variables (N=232)** | **Strongly Disagree**  **Value, n (%)** | **Disagree**  **Value, n (%)** | **Neutral**  **Value, n (%)** | **Agree**  **Value, n (%)** | **Strongly Agree**  **Value, n (%)** |
| --- | --- | --- | --- | --- | --- |
| I think I would like to use the Health4Life app frequently | 22 (9.5) | 42(18.1) | 112 (48.3) | 36 (15.5) | 20 (8.5) |
| I found the Health4Life App unnecessarily complex | 29 (12.5) | 77 (33.2) | 96 (41.4) | 24 (10.3) | 6 (2.6) |
| I thought the Health4Life app was easy to use | 12 (5.2) | 21 (9.1) | 75 (32.3) | 78 (33.6) | 46 (19.8) |
| I think that I would need the support of a technical person to be able to use the Health4Life app | 70 (30.3) | 65 (28.1) | 63 (27.3) | 21 (9.1) | 12 (5.2) |
| I found the various functions in the Health4Life app were well integrated | 13 (5.6) | 11 (4.7) | 126 (54.2) | 62 (26.7) | 20 (8.6) |
| I thought there was too much inconsistency in the Health4Life app | 27 (11.6) | 63 (27.2) | 109 (47.0) | 19 (8.2) | 14 (6.0) |
| I would imagine that most people would learn to use the Health4Life app very quickly | 9 (3.9) | 9 (3.9) | 87 (37.5) | 88 (37.9) | 39 (16.8) |
| I found the Health4Life app cumbersome to use | 15 (6.5) | 33 (14.2) | 143 (61.6) | 24 (10.3) | 17 (7.3) |
| I felt very confident using the Health4Life app | 8 (3.4) | 17 (7.3) | 85 (36.6) | 82 (35.3) | 40 (17.2) |
| I needed to learn a lot of things before I could get going with the Health4Life app | 36 (15.5) | 70 (30.2) | 92 (39.7) | 24 (10.3) | 10 (4.3) |

This is a Multimedia Appendix to a full manuscript published in the J Med Internet Res. For full copyright and citation information see http://dx.doi.org/10.2196/25513
